# A Method for Using Player Tracking Data in Basketball to Learn Player Skills and Predict Team Performance

**DOI:** 10.1371/journal.pone.0136393

**Published:** 2015-09-09

**Authors:** Brian Skinner, Stephen J. Guy

**Affiliations:** 1 Fine Theoretical Physics Institute, University of Minnesota, Minneapolis, 55455 MN, United States of America; 2 Massachusetts Institute of Technology, Cambridge, 02139 MA, United States of America; 3 Department of Computer Science, University of North Carolina at Chapel Hill, 27599 NC, United States of America; 4 Department of Computer Science and Engineering, University of Minnesota, Minneapolis, 55455 MN, United States of America; Queen’s University Belfast, UNITED KINGDOM

## Abstract

Player tracking data represents a revolutionary new data source for basketball analysis, in which essentially every aspect of a player’s performance is tracked and can be analyzed numerically. We suggest a way by which this data set, when coupled with a network-style model of the offense that relates players’ skills to the team’s success at running different plays, can be used to automatically learn players’ skills and predict the performance of untested 5-man lineups in a way that accounts for the interaction between players’ respective skill sets. After developing a general analysis procedure, we present as an example a specific implementation of our method using a simplified network model. While player tracking data is not yet available in the public domain, we evaluate our model using simulated data and show that player skills can be accurately inferred by a simple statistical inference scheme. Finally, we use the model to analyze games from the 2011 playoff series between the Memphis Grizzlies and the Oklahoma City Thunder and we show that, even with a very limited data set, the model can consistently describe a player’s interactions with a given lineup based only on his performance with a different lineup.

## Introduction

Numerical evaluation of basketball players has long been based on box score statistics. Such evaluations, by nature of the limited data set from which they draw, generally center around a player’s contributions to the five positive statistics—points, rebounds, assists, steals, and blocks—while neglecting more nuanced aspects of the player’s value, such as his/her ability to make high quality (non-assist) passes, or set good screens, or rotate effectively on defense. These less easily quantifiable aspects of a player’s performance are traditionally evaluated only qualitatively, informed by the intuition of a coach or analyst who has spent a significant amount of time watching the players perform.

This distinction between quantifiable and non-quantifiable player skills may be on the verge of disappearing. Beginning in the 2013–2014 season, all thirty arenas of the National Basketball Association (NBA) contain a system of cameras and tracking software that allow the spatial coordinates of all players and the ball to be recorded and processed digitally [1, 2]. In this way essentially every aspect of the game is made accessible for quantitative analysis—every pass, screen, and defensive rotation can in principle be analyzed quantitatively by those with access to this “player tracking” data.

Thus far, publicly-available studies using player tracking data have largely focused on augmenting or refining the use of conventional statistics. For example, recent studies have examined the effect of a defender’s proximity on shooting percentage [3], broken down shooting percentage based on how many dribbles are taken before the shot [1], characterized the effect of defender proximity on shooting percentage [4], and examined the dependence of rebound rate on spatial location [5]. These studies are certainly illuminating, and they suggest significant improvements that can be made to the conventional statistics by which players are evaluated. Recently, however, researchers have begun to consider that the usefulness of player tracking data may go well beyond creating or augmenting statistical descriptors of individual players, and may in fact catalyze a fundamental change in the way we think about the structure of basketball offense and defense [6, 7]. In this spirit, we consider here a similarly ambitious use for player tracking data.

Recent studies have proposed the idea that describing a basketball offense is essentially a network problem [8], in the sense that each possession progresses from a well-defined starting point toward a well-defined goal through a particular sequence of intermediary states. In this description, a “node” in the “offensive network” is a particular arrangement of the players and the ball within the offense, and a “link” in the network is the set of ball and player movements that are necessary to bring the offense from one node to another. The problem of optimizing the performance of the offense, then, can be seen as equivalent to optimizing the flow of possessions through the offensive network [8–10]—the offense should move as efficiently as possible from the first node (the inbounds pass) to the last (a made shot).

The power of this network description becomes apparent when one imagines coupling it with the full player tracking data set. Indeed, player tracking data allows one to reconstruct directly how the offense progresses from the inbounds pass to a made basket. The success rate of each step along the way provides information about the effectiveness of links in the network, which in turn are a reflection of the skill levels of the players involved. Thus, by “watching” the performance of the offense with player tracking data, it should be possible to learn the skill levels of the players involved, including any skills that affect the team’s offensive performance. Once these skills are known, one can predict quantitatively how the offense will perform when called upon to run different plays or to substitute different players whose skills are also known.

In this paper, we suggest a method for achieving these goals using player tracking data. We first outline generally how to build a network model of the offense that relates player skills to the effectiveness of a given play, and we provide a specific, simplified example of such a model. We then discuss the process of learning player skills from player tracking data, and we show that in general these skills cannot be measured directly from the data due to a lack of information about which play a team is attempting at the moment of a failed offensive sequence. Nonetheless, we show that this issue can be addressed using an iterative procedure based on Bayesian inference. The effectiveness of this procedure is then demonstrated by means of a test on simulated data. Finally, we use our simplified network model to examine hand-recorded data from the 2011 playoff series between the Oklahoma City Thunder and the Memphis Grizzlies. We show that, despite a very limited data set, we are able to correctly predict a player’s interactions with a particular lineup based on data from his performance with a different lineup.

In the following Results section, we present our method and examine one specific implementation of it using simulated and real data. The Discussion section briefly outlines some limitations and possible generalizations of our work. Finally, the Methods section describes details of the collection and processing of our NBA data.

## Results

### 1 Constructing a network description of basketball

In order to create a network model of a basketball offense, one must first define the set of nodes in the network. These nodes constitute unique states of the offense—i.e., the positions of all offensive players and the ball. For example, one can define the nodes by dividing up the court into discrete regions and then using the set of all possible locations of all five players and the ball to identify each node, so that every time a player moves from one region of the court to another the offense transitions from one node to another. (In principle, one can also incorporate the *velocity* of each player in the definition of nodes.) One could also employ a more course-grained definition of nodes, for example by neglecting the positions of off-ball players or by using a fine spatial grid for some players and a course grid for others. (In Ref. [11] it was suggested that such nodes could be defined based on the spatial locations where players tend to exhibit “bursts” of acceleration.) In the model described below in Sec. 3, we present a simplified example of such a network wherein each node is identified only by which player holds the ball and whether that player’s position is “high” or “low”.

Once a set of nodes is defined, one can take one of two approaches to describing the flow of possessions through the offensive network. The simpler approach is to view players as “random agents”, characterized only by their tendencies to move the offense in one way or another [12–14]. Under this description, the transition rates of the offense from one node to another can be measured directly from the data, and there is no need for the more advanced statistical inference algorithms that we describe in Sec. 3. This “random agent” description, while it may be useful for describing sports like soccer or hockey where there is generally no playbook from which the team operates, is unlikely to accurately describe professional basketball, where the progression of the offense is influenced both by the skills of the players and by the direction of the coach. A complete description of the random agent model is therefore confined to Appendix A.

The alternative to the random agent model can be called the “coached agent” model, which draws a distinction between the success rates of the various attempted transitions of the offense (“links”), which depend on the skill levels of the players involved, and the rates at which the plays are attempted, which depend on the coach and on the team’s overall offensive philosophy. A full description of a coached agent offense, then, involves knowing the set of all player skills and the rate at which each link in the network is attempted. If this information is known, then in principle the offense is completely described, and the effectiveness of the offense can be predicted under any number of hypothetical variations, including swapping one player for another or changing the team’s set of attempted plays. (In other words, a description of an offense of “coached agents” allows one to address the problem of optimal play calling.) In Sec. 5 we comment briefly on possible hybrids between the “coached” and “random” agent models.

**Fig 1:**
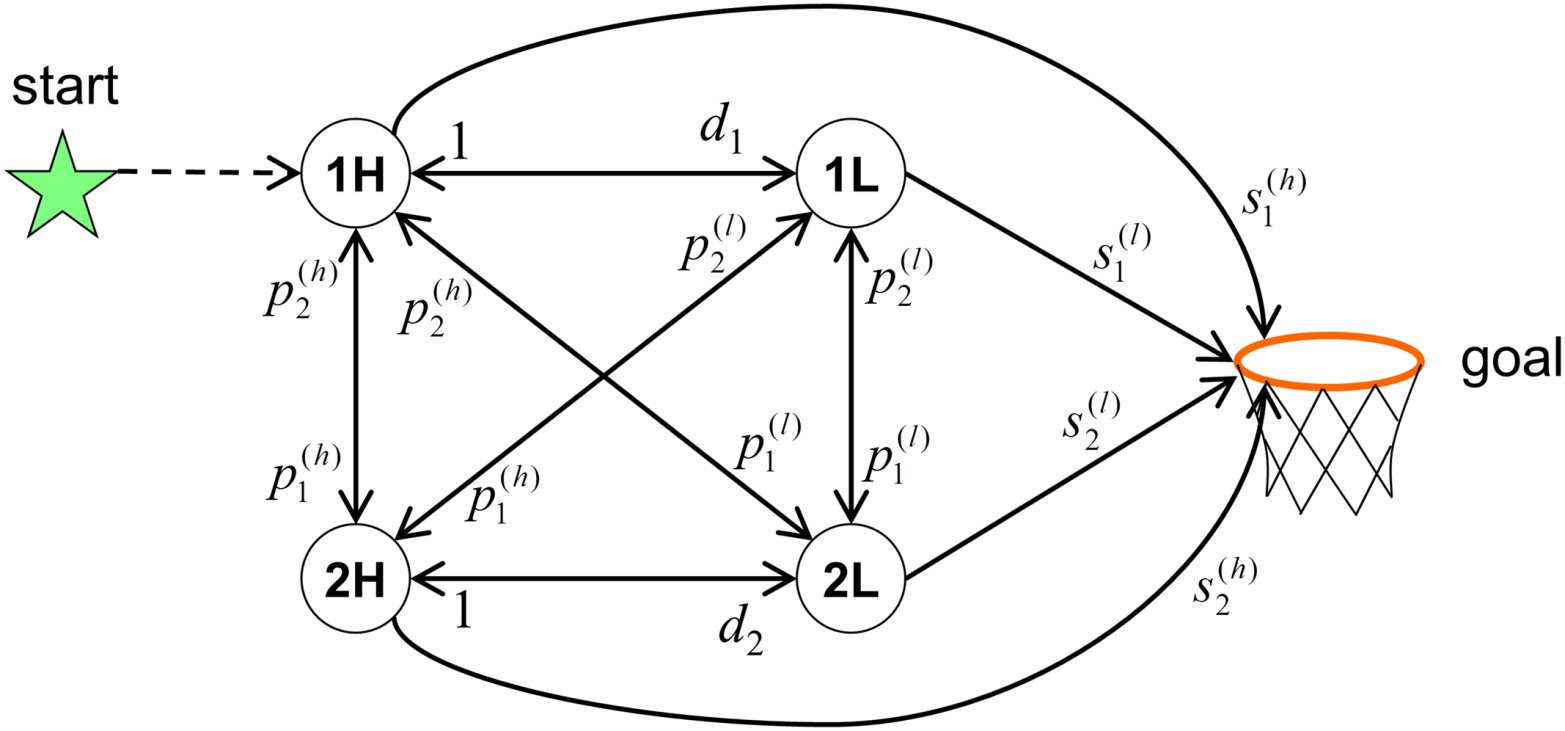
Diagram of the high/low model of a basketball offense. For clarity, the diagram shows only two of the five offensive players; the full diagram has 11 nodes (including the goal) and 110 links. Each link is labeled by its corresponding success rate.

In the coached agent model, one must provide not only a definition of the nodes of the offense, but also an analytical definition of how the success rates of the links between these nodes depend on the skill levels of the players involved. For example, if node *a* describes a state where the point guard has the ball on the wing and node *b* describes a state where the center has the ball in the low post, then the success rate *f*_*ab*_ of link *ab* must depend on the point guard’s skill in making the entry pass. More generally, for each possible link *ij* one needs to define a formula *f*_*ij*_(*S*) that relates the set of all player skills, denoted *S*, to the probability of the offense successfully transitioning from node *i* to node *j*, given that the transition is attempted (in general, *f*_*ij*_ ≠ *f*_*ji*_). In this way, the definition of all success rates {*f*_*ij*_} also involves the quantitative definition of all player skills that are relevant to the offense. One specific definition of player skills and success rates is discussed in Sec. 2 and diagrammed in Fig 1. For those links *ij* that involve a shot attempt—i.e., where the node *j* represents the goal—*f*_*ij*_ is best defined as the expected number of points scored by the shot attempt (so that the value of *f*_*ij*_ depends on whether the shot taken is a 2-point or a 3-point attempt).

Once the formulae *f*_*ij*_(*S*) are defined, one can predict the expected number of points scored per possession by the offense, *F*, by summing over all possible attempted plays *α*, weighted by the probability *P*_*α*_ that the team will attempt them during a given possession:

$$\begin{array}{c} F=\sum_{\text{all}\;\text{plays}\;\alpha }P_{\alpha }\prod_{\text{all}\;\text{links}\;ij\in \alpha }f_{ij}\left(S\right). \end{array}$$ (1)

In this paper, we use the term “play” to mean “a sequence of moves designed by the offense to produce a shot attempt.”

Of course, it is important to note that the apparent skill levels of the players, which define the success rates *f*_*ij*_ of different attempted transitions, depend not only on the skills of the offensive players but on the quality of the *defense* being faced. In this sense any player skill is understood to be defined relative to a given defense. When considering the performance of a player over an entire NBA season, for example, such skills are naturally defined relative to the league-average defense. One could also imagine considering a player’s performance against a specific opponent, and thus defining the player’s skills relative to that particular matchup.

In Sec. 4 we show how player tracking data can be used to learn the set of all player skills, so that the effectiveness *F* of the offense can be evaluated for any set of play frequencies *P* ≡ {*P*_*α*_} and any combination of players. But first we outline a simple model of a basketball offense, which we call the “high/low” model, that will serve as a proof of concept for the network approach advocated in this paper.

### 2 The high/low model

In the previous section we outlined the general ingredients necessary for creating a network model of a basketball offense. In this section we introduce one specific example of such a model, which we dub the “high/low” model. In this model, the state of the system is considered to be entirely characterized by which player holds the ball and whether that player’s position is “high”—far from the basket—or “low”—close to the basket. We set the cutoff between high and low using the perimeter of the free throw lane (the “key”). For example, if the point guard holds the ball at the top of the key, this state is labeled “1 high”, or “1H”; if the shooting guard has the ball in the key this is labeled “2 low”, or “2L”; etc. (Following the convention of basketball coaching, the five offensive players on the court are numbered 1—5, with 1 being the point guard, 2 the shooting guard, 3 the small forward, 4 the power forward, and 5 the center.) The nodes of this network are shown as bold, circled characters in the diagram of Fig 1, which for visual simplicity depicts a network with only two players.

Within this model, we further assume that the success rate of any given link depends only on the skills of the player holding the ball. Specifically, the success rate of a pass by player *i* to another player in a “high” state is defined to be equal to player *i*’s high passing ability $p_{i}^{\left(h\right)}$; the success rate of a low pass is denoted $p_{i}^{\left(l\right)}$; the success rate of a high shot is $s_{i}^{\left(h\right)}$; and the success rate of a low shot is $s_{i}^{\left(l\right)}$. The probability of player *i* successfully moving from high to low is defined as the player’s “driving ability” *d*_*i*_. For simplicity, we take the success rate of a move from low to high to be equal to unity.

Within the model diagrammed in Fig 1, the quality of the offense is completely characterized by the shooting skills $\left\{s_{i}^{\left(h\right)},s_{i}^{\left(l\right)}\right\}$, passing skills $\left\{p_{i}^{\left(h\right)},p_{i}^{\left(l\right)}\right\}$ and driving skills {*d*_*i*_} of each player. Thus, one can completely determine the expected number of points scored by the team for a given use of plays {*P*_*α*_} by performing the sum over all plays denoted by Eq (1).

Of course, one can easily imagine more involved models for the offense than the high/low model presented here; for example, one could introduce a more finely resolved definition of player positions or define success rates in terms of multiple players’ activies rather than just that of the ball handler. Nonetheless, we show in Sec. 5 that even this very simple model can provide a basis for describing a player’s skill set and predicting its interaction with the skills of other players, even using a very limited data set.

### 3 A statistical inference algorithm for learning player skills

Unlike in the “random agents” model detailed in Appendix A, for the “coached agents” model that is the main subject of this paper, one cannot, in general, measure player skills directly from player tracking data. This is because player tracking data provides only a sequence of plays as they are performed by the players, and does not give any information about the team’s original intentions (i.e., the play as diagrammed by the coach). Consider, for example, the problem of determining a player’s passing skill in the high/low model. A typical possession within this model might progress from the state 1H to the state 3H to a turnover (denoted, for brevity, 1H → 3H → T). But in this sequence it is not clear how the turnover by the small forward should be interpreted. Was the turnover the result of a failed high pass? A failed low pass? A failed drive? In order to determine how to assign blame for this turnover among the player’s various skills, one needs to know something about which plays the team tends to attempt. Thus, the frequencies of play calls {*P*_*α*_} must be inferred simultaneously with the player skills. In this endeavor one can employ a statistical inference algorithm, such as the one we outline below.

Generally speaking, inference algorithms are used to infer the parameters of a statistical model in cases where there are latent variables, which cannot be measured directly from the data [15]. In our case, the relevant model is the network description of a basketball offense (for example, the high/low model), the parameters are the player skills, and the latent variables are the frequencies with which different plays are attempted by the team. The data from which model parameters are to be estimated consists of an ordered list of observed states of the offense for each possession (for example, data from one possession might read 1H → 5L → 2 points). A successful inference scheme should provide a way to simultaneously estimate the set of player skills *S* and the set of play frequencies *P* from this data using an iterative procedure. One particularly straightforward implementation of this procedure is as follows.

First, an initial guess is made for *P*—that is, for the value of the frequency with which all plays are attempted. For example, below we use the guess where all play frequencies are taken to be identical. One should also assume a prior distribution for each of the skill values. Here, as an example, we use the uninformed “flat” prior, which assumes that all skill values are drawn with equal probability from their entire domain. These initial guesses for *P* and *S* are refined over time, and should not be reflected in the final inferred values of *S* for a large enough dataset. This can be verified explicitly by running the algorithm with different initial guesses.

It is important to note that, in principle, the number of elements in *P* should be very large in order to account for all possible plays that could be run by the team. In practice, however, it is sufficient to approximate the offense by some finite number *N*_*p*_ of plays that tend to be attempted. These can be inferred from the data by collecting the *N*_*p*_ most commonly seen plays by the offense that end in a shot attempt. The effect of increasing the size of the play set *N*_*p*_ is examined in Appendix B.

Once initial guesses have been made for *P* and *S*, one can estimate values for each player skill *S* using the following algorithm. First, one should count the total number of attempted and failed demonstrations of each skill in the dataset. For possessions ending in a shot attempt, this counting is straightforward: all steps before the shot attempt represent one successful demonstration of the corresponding skill (for example, the passing/driving skill of the ball handler), and the shot attempt represents one attempt at a shot (either successful or failed). For posessions ending in a turnover, however, the failed play can be counted fractionally toward a number of different skills, with the count being weighted by the probability that a given movement of the offense was being attempted. In particular, one should estimate the probability Prob(*α*∣*k*) that the observed sequence *k* was the result of an attempt to run a given play *α*, and then count each step of the observed sequence as a number Prob(*α*∣*k*) of attempts to run the corresponding step of play *α*. The probability Prob(*α*∣*k*) can be estimated using Bayes’ rule:

$$\begin{array}{c} \text{Prob}\left(\alpha |k\right)=\frac{\text{Prob}\left(k|\alpha \right)P_{\alpha }^{\left(e\right)}}{\text{Prob}(k)}. \end{array}$$ (2)

Here, Prob(*k*∣*α*) is the *a priori* probability that an attempt to run play *α* will result in the observed sequence *k*; this probability is a function of the skills of the players involved. The value $P_{\alpha }^{\left(e\right)}$ is the previously estimated probability that the team will attempt to run play *α* on a given possession, and Prob(*k*) is the total probability that a randomly chosen possession will follow the sequence *k*: $\text{Prob}(k)=\sum_{\alpha^{\prime }}\text{Prob}(k|\alpha^{\prime })P_{\alpha^{\prime }}^{\left(e\right)}$.

Once the number of failed and attempted skill demonstrations have been tallied, one can use them to produce updated estimates for the skill values, *S*. More specifically, these numbers are used to produce posterior distributions for each skill value. This is described in detail for the high/low model in Appendix C. In the limit of a very large dataset, the posterior distributions have small variance and the estimated skill value is generally defined by the ratio of the number of successful skill demonstrations to the total number of attempted skill demonstrations (*i.e*., the success rate of the particular skill). We emphasize, however, that these numbers cannot, in general, be read directly from the dataset. As discussed above, making an estimate of skill values requires one to determine how blame should be assigned for failed play sequences, and this necessitates the use of a statistical inference scheme.

Finally, once updated estimates for the skills *S* have been obtained, one can update the estimate for the play frequencies $\left\{P_{\alpha }^{\left(e\right)}\right\}$ according to

$$\begin{array}{c} P_{\alpha }^{\left(e\right)}=\frac{\sum_{k}\text{Prob}\left(k|\alpha \right)}{N}, \end{array}$$ (3)

where ∑_*k*_ denotes the sum over all sequences in the dataset, and *N* is the total number of such sequences.

The process outlined in the previous three paragraphs gives improved estimates of the player skills *S*, as defined by the posterior distribution for each skill (see Appendix C), and for the team play frequencies *P*, starting with an initial guess. This process can be repeated multiple times, replacing $P_{\alpha }^{\left(e\right)}$ and *S* by their newly-estimated values after each iteration, until all the elements of *S* and *P* converge. In practice, ∼ 10 iterations are generally sufficient to produce convergence, and 100 possessions (about one game’s worth) can be processed within a few seconds.

### 4 Test on simulated data

In order to verify that the method outlined in Sec. 3 can consistently learn player skills, we tested our inference algorithm on simulated data from the high/low model. Specifically, we wrote a short simulation of a team of five players with arbitrarily-chosen skills running plays from a playbook of twenty plays. The play frequencies *P* were also randomly chosen, and 5000 possessions were simulated—this corresponds to 82 games’ worth of data (a full NBA season) for a lineup that plays together roughly 30 minutes per game. This data was processed using the algorithm described in Sec. 3 in order to infer the assigned traits without any prior input regarding their true values. The inferred values were then compared to the ones input to the simulation in order to assess the accuracy of the algorithm.

**Fig 2:**
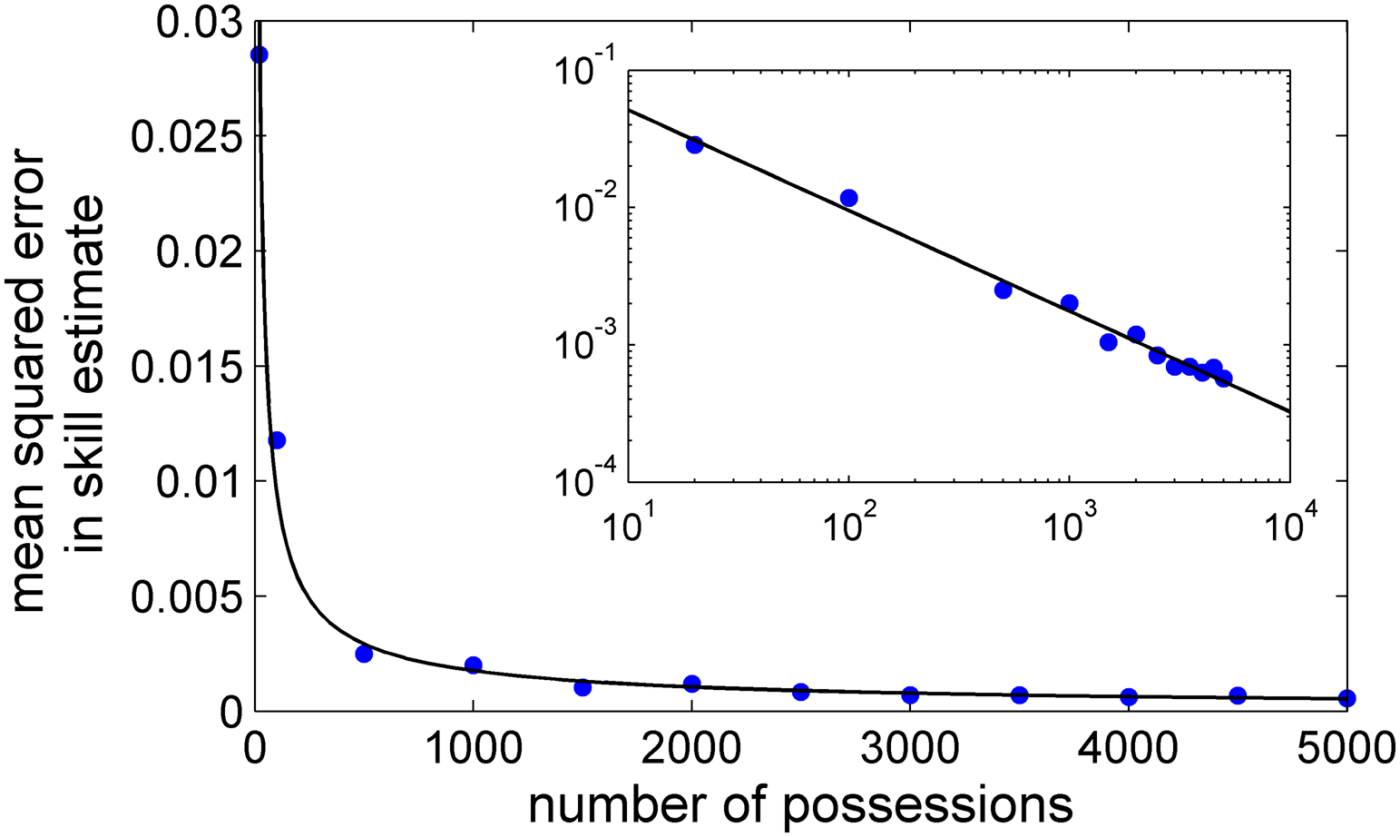
The mean squared error in the inferred value of player skills for a simulated high/low offense as a function of the number of possessions analyzed. The curve is a power-law fit. The inset shows the same data plotted in logarithmic scale.

Fig 2 shows the resulting mean squared error in the inferred player skills as a function of the number of possessions analyzed. The results suggest that after a season’s worth of data the player skills are known to within a few percent, and that the inferred value of skills generally converge to the actual values after a few hundred possessions. This analysis uses *N*_*p*_ = 20; results at other values of *N*_*p*_ are given in Appendix B.

### 5 The 2011 OKC/Memphis NBA Playoff series

In Sec. 4 we showed that our statistical inference algorithm can correctly infer the skill levels of players in a simulated offense. Given this success, the prospect of using the algorithm to learn the traits of actual NBA players is naturally enticing. A successful inference scheme, coupled with a suitably sophisticated network model, would allow one to learn the skill levels of NBA players from player tracking data and thus predict how the team’s effectiveness would be affected by any number of changes, including player trades, substitutions, or altered play calling.

Unfortunately, player tracking data is not yet available in the public domain. Thus, we devised the following basic test of our general strategy. In order to obtain some “poor man’s player tracking data”, we recorded by hand data corresponding to 401 possessions from the 2011 playoff series between the Oklahoma City Thunder (OKC) and the Memphis Grizzlies. This data is described in more detail in the Methods section. Possessions were then sorted according to which lineup the team had on the floor. For example, the Memphis starting lineup of Conley-Allen-Young-Randolph-Gasol played 84 possessions together during the first three games, while the OKC starting lineup of Westbrook-Sefolosha-Durant-Ibaka-Perkins played 111 possessions; we refer to these as “lineup 1” for each team. Memphis’s second-most-used lineup was Conley-Mayo-Battier-Randolph-Gasol, while for OKC it was Westbrook-Harden-Durant-Collison-Ibaka; these we denote “lineup 2”.

One particularly exciting feature of our method is its ability to potentially predict how a given player will contribute to an offense based on his performance in a different offense and surrounded by different teammates. We tested this feature by examining how well the inferred skills for a player in one lineup can predict that player’s skills in a separate lineup. For the remainder of this section we focus our analysis on the point guards Conley and Westbrook, for whom the data was most robust, given their centrality to the movement of the offense. We used our inference algorithm to infer the skills of Conley and Westbrook using only the data corresponding to lineup 2, and then we checked whether these inferred skills constituted an accurate prediction of the skills exhibited by the players during their performance in lineup 1.

**Fig 3:**
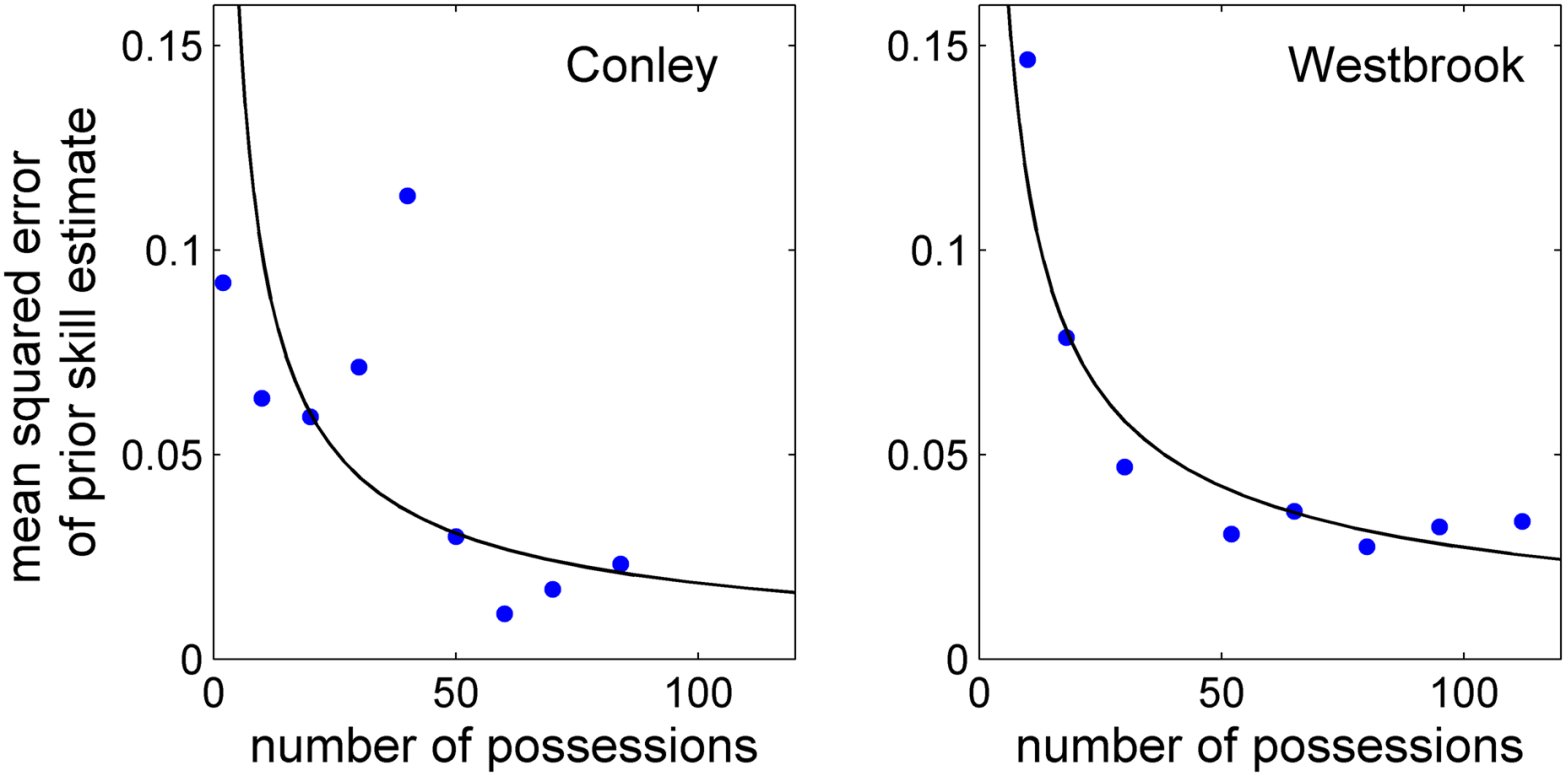
The mean squared difference between the skills inferred from lineup 1 and the skills inferred from lineup 2 as a function of the number of possessions analyzed from lineup 1. As more data is analyzed, the inferred skills for both Conley and Westbrook converge toward the prior estimate based on their performance in lineup 2.

Fig 3 shows that, indeed, the skills inferred from lineup 2 serve as an accurate input for predicting the players’ performance in lineup 1, even though each player has a significantly different role in lineup 2 as compared to lineup 1. As more data is included from lineup 1, we find that the two estimates come closer and closer. This result suggests that, despite the extremely limited nature of the data being analyzed and the parsimony of the high/low model, our approach has the ability to predict quantitatively how a player will perform given a different set of teammates and a different offensive role. For example, Westbrook’s effectiveness in lineup 1 can be correctly inferred from his noticeably more aggressive performance in lineup 2—in lineup 2, Westbrook produced a shot attempt or a turnover on 37/86 of his team’s possessions, or 43%, while in lineup 1 that ratio was only 33/112, or 29%. A table containing the inferred skills of both Conley and Westbrook is given in Appendix D along with some relevant discussion.

A more stringent test of our approach would be to use the inferred skills for a group of players to predict their offensive performance after a significant lineup change (say, after one of the players is traded for another) or a change in offensive philosophy (say, after a new coach is acquired). Unfortunately, our very limited data set does not allow us to perform such a test at present.

## Discussion

In closing, we would like to emphasize that the goal of this paper is not to report a particular empirical finding or to promote a particular set of metrics and diagrams for basketball analysis, but rather to suggest an exciting and novel strategy for taking advantage of a revolutionary data source. The network-based strategy outlined here offers the potential for a new degree of predictive power for analysts, managers, and coaches in sports. Namely, it comprises a tool for learning, in a completely automated and unbiased way, the skill levels of different players and for predicting quantitatively how those players will perform together as a team. Such a tool has the potential to have a significant effect on coaching, scouting, and personnel decisions in professional sports [2].

Of course, the real impact of the approach suggested here awaits its application to the actual player tracking data. Under the influence of such data, the simple high/low model will likely be replaced by richer models, including perhaps models that incorporate a finer spatial grid or a continuous description of spatial positions. Such detailed models may require one to adapt more advanced techniques from spatial statistics (as, for example, in Refs. [15, 16]).

We also note that, while our analysis has focused on basketball, our method can be equally applied to any sport that can be modeled as a network [17]. Our definition of a “network” as a sequence of states connected by skill-based transitions is extremely general, and can readily be extended to other individual or team sports. For some situations it may be expedient to create a hybrid between the “coached agent” and “random agent” models we have described here and in Appendix A, in which players are described as making autonomous, random decisions within some limited aspects of the offense, while other aspects are dictated by the coach or team strategy. While this hybrid would require some further extensions of the formal methods presented in Secs. 1 and 3, there is no reason to think that these extensions would be prohibitive.

One can also notice that, while this paper has focused on optimizing the performance of an *offense*, the entire analysis can be recast in terms of *defense* as well. For a defensive team, the goal is to minimize the effectiveness of the opposing offensive network. Thus, one can define a player’s defensive skills in terms of how well they prevent certain transitions in the opposing offense. In this way the entire analytical method can be used directly on the defensive end, and the effectiveness of an interacting “team defense” can be quantified. The full quality of the team, then, is the difference between the effectiveness *F* of the offensive network [given by Eq (1)] and the entirely analogous effectiveness of the defense.

In closing, we look forward to future work that can apply the approach we have advocated here to a large corpus of player tracking data. Such work will likely spur the development of new network-style models and refined statistical inference algorithms, but we remain confident that the general strategy we have described can be successful if given sufficient attention and the full power of many games worth of player tracking data. If this is indeed the case, then perhaps the network-based description of basketball will provide a suitably revolutionary application for a revolutionary new data source.

## Methods

The data analyzed in Sec. 5 was recorded by hand from the first three games of the 2011 playoff series between the Oklahoma City Thunder and the Memphis Grizzlies. Footage for the games was provided by Synergy Sports (www.mysynergysports.com). For each possession, defined as the interval of time in which one team continuously has possession of the ball, the sequence of offensive states was recorded as defined by the “high/low” model described in Sec. 2. Specifically, the state of the offense at a given instant was labeled according to which player (labeled 1–5) had the ball and whether that player was outside the key (“high”, or H) or inside the key (“low”, or L). A total of 780 such sequences were recorded, 401 for Memphis and 379 for OKC.

Each sequence was identified with the five-man lineup on the floor at the time it was performed. The two most common lineups for each team are analyzed in Sec. 5. Sequences that did not correspond to these lineups were not included in the analysis.

## Appendix A The “Random Agents” model of a basketball offense

In the main body of this paper, which focuses on the “coached agent” model, a distinction is drawn between players’ *intentions* and players’ *abilities*. In other words, the team’s use of plays *P* is considered a separate input to the offense from the players’ skills *S*. This distinction allows one a much enhanced ability to predict the offense’s performance. However, it also greatly increases the number of variables necessary to describe the offense and requires the use of inference algorithms, as described in Sec. 3. A much simpler approach is to abandon the distinction between skills and intentions and talk only in terms of players’ *tendencies*. This approach amounts to assuming that the movement of the offense starting at any particular node is a random decision that is determined only by which players are on the court. In other words, in the “random agent” model the offense is described as a Markov chain, jumping randomly from one node to another in search of the goal [12–14].

While the random agent description offers significantly less predictive power than the coached agents model—it becomes impossible to describe how a team’s performance will be different under a different offensive scheme, or how a player’s decisions will change with a different set of teammates—it offers the advantage of being much simpler to implement while still capturing some effect of the interaction between players’ respective skill sets [18]. While we remain doubtful that it can be implemented to great success in professional basketball, it is easy to imagine that the random agent model can be beneficial in soccer, hockey, or other sports where teams don’t tend to use pre-established plays.

In the random agent model, the effectiveness of a team of players can be determined as follows. First, one should expand slightly the set of nodes of the offense to account explicitly for the possibility of different possession-ending outcomes. For example, in basketball one would define separate nodes for “missed shot”, and “turnover”. Each pair of nodes *ij* has a particular transition rate *r*_*ij*_ that can in principle be measured directly from the data. For example, if *h* is the node that represents a given player holding the ball in the high post and *t* represents a turnover, then *r*_*ht*_ is the fraction of the time that the player turns the ball over when given the ball in the high post. For any possession-ending outcome *e*, the rate *r*_*ei*_ is zero for all nodes *i* except *i* = *e*, for which *r*_*ei*_ = 1. In technical language, one can say that *e* is an “absorbing state”. (A missed shot *m*, generally speaking, cannot be classified as a possession-ending outcome, since some missed shots produced offensive rebounds. Thus, *r*_*mm*_ < 1, and this represents the probability of a player’s shot not being rebounded by his own team.) If all transition rates *r*_*ij*_ are known, then one can assemble them into a “propagation matrix” *U*, whose elements *U*_*ij*_ = *r*_*ij*_. This matrix describes the evolution of the offense during one step.

To determine the expected offensive output of the team, one should know the average initial state of the offense, which can be described as a vector *y* whose elements *y*_*i*_ denote the probability that the team starts its offense at node *i*. For example, if the offense always starts with the ball in the point guard’s hands, then *y* is a vector whose elements are all zero except for that element corresponding to the node 1H (or its equivalent), which has *y*_1H_ = 1. The product *Uy* produces a vector that describes the expected state of the offense after one step. The state of the offense after many steps is given by *U* ^*n*^ *y*, with *n* → ∞; here, *U* ^*n*^ denotes the matrix *U* multiplied by itself *n* times. (The matrix *U* ^∞^ is analogous to the “S-matrix” in quantum mechanics, which dictates the evolution of a system from its initial state.) Thus, if *g* corresponds to the “goal” node indicating points scored, then the expected output of the offense is

$$\begin{array}{c} F=\lim_{n\to \infty }{\left(U^{n}y\right)}_{g} \end{array}$$ (4)

Here, the subscript *g* indicates the *g*th component of the vector *U* ^*n*^ *y*.

Thus, the expected offensive output can be evaluated if the tendencies *r*_*ij*_ are known for all players in the offense. Interactions between player skills arise in the way that different rates *r*_*ij*_ are chained together sequentially in moving the ball from the initial state *y* to the goal *g*. Within this model, one can imagine swapping out one player for another, so that the corresponding transition rates *r*_*ij*_ are modified, and then evaluating Eq (4) to see whether the team’s overall performance has improved or declined.

## Appendix B Effect of play set size

In Sec. 3 we showed how a statistical inference algorithm can be used to infer player skills. This inference procedure requires one to make an estimate of which plays the team tends to attempt, which allows one to construct the set of play frequencies *P*. In principle, there is an enormous number of potential plays that a team can attempt, which is limited only by the finite duration of the shot clock. In any reasonable offensive model, trying to include every possible play that the offense can attempt makes *P* so large that it becomes impossible to deal with computationally. In practice, however, the vast majority of these possible plays will never be attempted. For example, the play 1H → 5L → 2L → 5L → 2L → 5L → 2L → goal has probably never been attempted in the history of professional basketball, much less occupied an importance place in a team’s playbook. Similarly, the great majority of sequences that are possible for the offense occur so rarely that they can be safely ignored by our algorithm. Instead, our strategy is to construct a finite set of possible plays that the team is likely to attempt by making a list of all possessions recorded in the data that end in a shot attempt and then choosing from this list the top *N*_*p*_ most frequently used.

**Fig 4:**
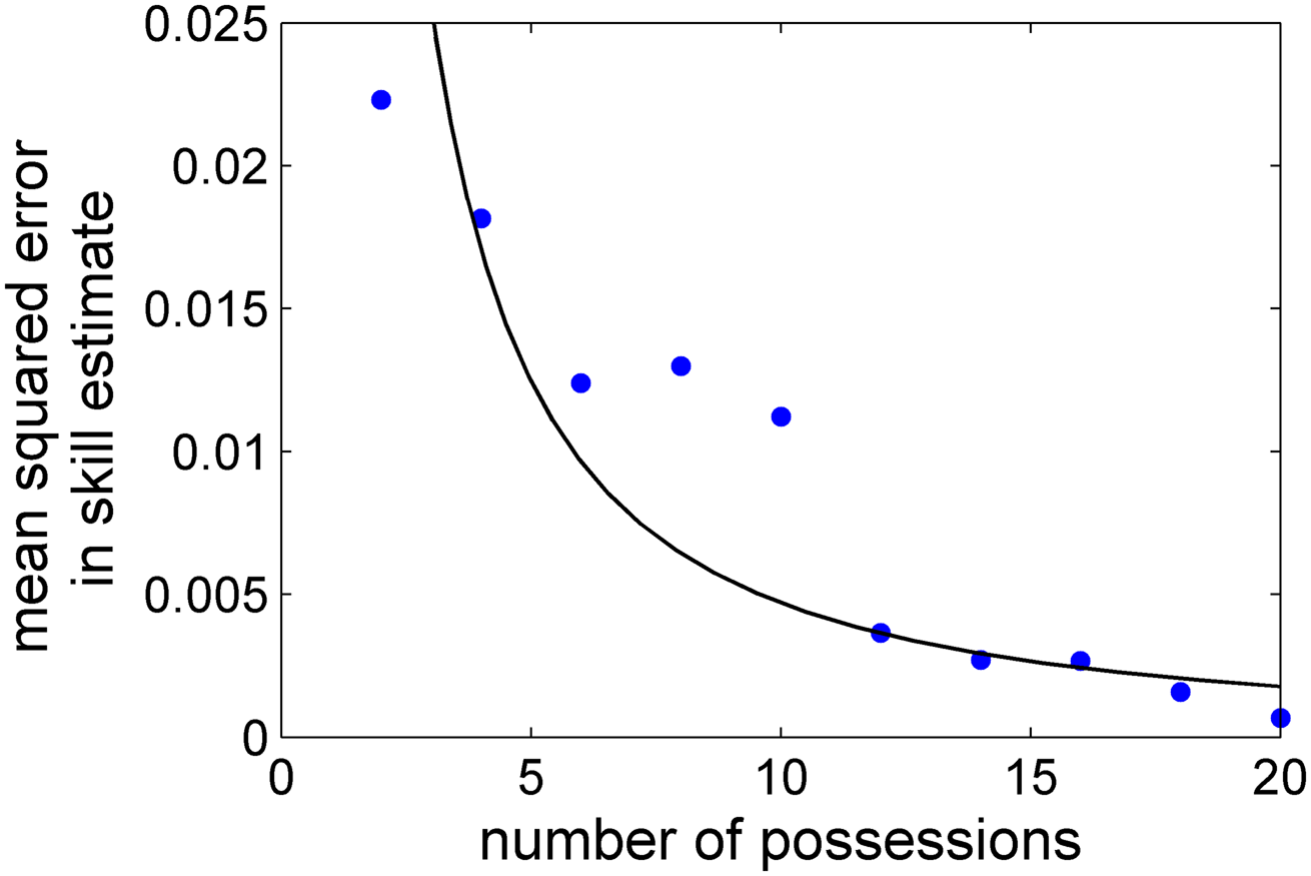
The mean squared error in the inferred value of player skills for a simulated high/low offense as a function of *N*_*p*_, the number of plays included in the set of possible team plays. 10,000 possessions are analyzed for each value of *N*_*p*_. The curve is a power-law fit.

Clearly, if the number *N*_*p*_ of plays included is too small, the model will fail to accurately describe the offense and thus provide a poor fit to the data, while if *N*_*p*_ becomes too large one runs the risk of over-fitting the data and losing any predictive power. In order to see how the accuracy of the algorithm depends on the number of plays included in *P*, we evaluated the accuracy of the algorithm’s inferred skills as a function of *N*_*p*_, again using the simulated data described in Sec. 3. The result is shown in Fig 4, which generally suggests that the accuracy of the method converges fairly quickly as more potential plays are added to *P*. Given this result, we speculate that for the description of a real (professional) basketball offense it is likely sufficient for *P* to contain only a few dozen potential plays.

## Appendix C Prior and posterior distributions for the skill values in the high/low model

The goal of the inference algorithm presented in Sec. 3 is to arrive at estimates for each skill value in the offense. Equivalently, one can say that the algorithm is designed to use some set of data *D* to produce an accurate posterior distribution *f*_post_(*s*∣*D*) for a given skill *s*. This posterior distribution is related to the assumed prior distribution, *f*_prior_(*s*) by

$$\begin{array}{c} f_{\text{post}}\left(s|D\right)\propto \text{Prob}\left(D|s\right)f_{\text{prior}}\left(s\right). \end{array}$$ (5)

Here, Prob(*D*∣*s*) is the probability that a player with skill *s* will produce the observed data *D*. In this paper, we choose for the prior distribution *f*_prior_(*s*) a simple uniform distribution, which can be written in the notation of the beta distribution as *f*_prior_(*s*) = Beta(*s*;1,1). (Here, the final two arguments *a* and *b* of the function Beta(*s*;*a*, *b*) are the shape parameters of the beta distribution.)

In the first step of the inference algorithm described in Sec. 3, initial guesses for the set of play frequences *P* are used to arrive at estimates for the number of successful skill demonstrations, *n*_s_, and for the number of total attempted skill demonstrations, *n*_tot_. If the outcome of each step of each play is considered to be statistically independent, then the probability Prob(*D*∣*s*) is a binomial distribution: Prob(*D*∣*s*) = Binomial(*n*_s_;*n*_tot_, *s*). Here, the skill *s* is taken to be the probability of successful completion of a given step of a play (e.g., the passing or driving skill of a particular player, or the player’s shooting percentage). In this case, since the beta distribution is conjugate to the binomial distribution, the posterior distribution is also a beta distribution:

$$\begin{array}{c} f_{\text{post}}\left(s|n_{\text{s}},n_{\text{tot}}\right)=\text{Beta}\left(s;n_{\text{s}}+1,n_{\text{tot}}-n_{\text{s}}+1\right). \end{array}$$ (6)

More generally, in cases where one can make a more informed statement about the prior expectation for player skill values, the prior distribution can be taken to be *f*_prior_(*s*) = Beta(*s*;*α*_0_, *β*_0_) with some shape parameters *α*_0_, *β*_0_ > 0. Using the beta distribution as a prior allows one to write a simple expression for the posterior distribution:

$$\begin{array}{c} f_{\text{post}}\left(s|n_{\text{s}},n_{\text{tot}}\right)=\text{Beta}\left(s;n_{\text{s}}+\alpha_{0},n_{\text{tot}}-n_{\text{s}}+\beta_{0}\right). \end{array}$$ (7)

The corresponding mean skill value is then

$$\begin{array}{c} \left\langle s\right\rangle =\frac{n_{\text{s}}+\alpha_{0}}{n_{\text{tot}}+\alpha_{0}+\beta_{0}}, \end{array}$$ (8)

and the associated standard error is

$$\begin{array}{c} \sigma_{s}=\sqrt{\frac{\left(n_{\text{s}}+\alpha_{0}\right)\left(n_{\text{tot}}-n_{\text{s}}+\beta_{0}\right)}{{\left(n_{\text{tot}}+\alpha_{0}+\beta_{0}\right)}^{2}\left(n_{\text{tot}}+\alpha_{0}+\beta_{0}+1\right)}}. \end{array}$$ (9)

In the main text, when we refer to an “estimated skill value”, we generally mean 〈*s*〉 as given by Eq (8) with *α*_0_ = *β*_0_ = 1. Of course, as mentioned in Sec. 1, for shooting skills this probability is multiplied by the average number of points scored per made shot for the player in question, so that *s* (as, for example, in Table 1) represents the number of points scored per shot attempt.

**Table 1:** Inferred skill values for Mike Conley and Russell Westbrook based on their performance in the first three games of the 2011 Memphis-OKC playoff series. The skills are based on the high/low model, defined in Sec. 2 and Fig 1, and are listed together with ± one standard error [see Eq (9)].

Player	*s* ^(*h*)^	*s* ^(*l*)^	*p* ^(*h*)^	*p* ^(*l*)^	*d*
Conley	1.56±0.29	1.01±0.29	0.98±0.01	0.98±0.03	0.87±0.07
Westbrook	0.66±0.20	1.04±0.20	0.98±0.02	0.75±0.11	0.85±0.06

## Appendix D Inferred skills for Conley and Westbrook in the high/low model

In Sec. 5 we briefly described our results analyzing NBA games between the Memphis Grizzlies and the Oklahoma City Thunder in terms of the high/low model presented in Sec. 2. Table 1, below, shows the values of the player skills that were inferred for the point guards of both teams, Conley and Westbrook, against the opposing defense. Each listed skill corresponds to a weighted average between the values inferred from lineup 1 and lineup 2. The definition of each skill is provided in Sec. 2.

Based on the inferred values of *S* and *P*, Eq (1) predicts a nearly identical offensive efficiency *F* for the two teams’ starting lineups: *F*_OKC_/*F*_Memphis_ = 1.02. It is perhaps not surprising, then, that the series took seven games and four overtimes to decide.

One can notice from Table 1 that Conley’s high/low skills compare very favorably with Westbrook’s, rating essentially identical in all skills except for high shooting, where Conley rates significantly higher. This favorable comparison comes despite the fact that Westbrook is generally considered the better player. This discrepancy can likely be attributed to the very small data sample size—in a data set of three games, one “off game” by Westbrook or “hot” game by Conley would greatly alter their relative perceived value. (In Game 3, for example, Wesbrook shot only 7–22 from the floor and committed seven turnovers, while in Game 2 Conley show 8–10 from “high”, with three 3-pointers.) And, certainly, no one who watched Conley’s performance during these three games should be surprised that his apparent skill levels are very high.

On the other hand, there is perhaps an important dependency reflected in Table 1 that is not captured directly by our network description. Westbrook is generally called upon to carry a significantly larger offensive load than Conley, a fact that is reflected in the much larger percentage of OKC’s possessions that end with Westbrook taking a shot or committing a turnover: about 35%, as compared to only about 15% for Conley. In basketball analysis, it is generally accepted that larger usage rates for a given player are correlated with declining success rates, due primarily to the increased focus such a player receives from the defense. Such a relationship is called a “skill curve” [19], and under certain situations it can lead to surprising and counterintuitive phenomena in the offensive network [8]. Any dependencies of success rate on usage rate are not considered explicitly in this paper, but in principle they can be incorporated into the functional definitions of the network success rates *f*_*ij*_, which become functions not just of the player skills *S* but of the play usage rates *P* as well.

For models that do not make explicit consideration of such effects, one should be careful when making predictions about hypothetical lineups to ensure that each player’s usage rate in the new offense is not significantly different than the player’s usage rate in the data from which their skills are inferred. Otherwise, one can get spurious predictions based on unrealistically robust estimates of player skills.
